# Presence of Autoimmune Antibody in Chikungunya Infection

**DOI:** 10.1155/2009/840183

**Published:** 2009-11-25

**Authors:** Wirach Maek-a-nantawat, Udomsak Silachamroon

**Affiliations:** Department of Clinical Tropical Medicine, Faculty of Tropical Medicine, Mahidol University, 420/6 Ratchavithi Rd., Ratchathewee 10400, Bangkok, Thailand

## Abstract

Chikungunya infection has recently re-emerged as an important arthropod-borne disease in Thailand. Recently, Southern Thailand was identified as a potentially endemic area for the chikungunya virus. Here, we report a case of severe musculoskeletal complication, presenting with muscle weakness and swelling of the limbs. During the investigation to exclude autoimmune muscular inflammation, high titers of antinuclear antibody were detected. This is the report of autoimmunity detection associated with an arbovirus infection. The symptoms can mimic autoimmune polymyositis disease, and the condition requires close monitoring before deciding to embark upon prolonged specific treatment with immunomodulators.

## 1. Introduction

Chikungunya is a mosquito-borne arboviral infection. The virus can be epidemic and is endemic in Africa, India, the Indian Ocean islands, and Southeast Asia [1–3]. It has been reported sporadically from some provinces in Thailand [4], in 1988, 1991, and 1993, since its first detection in Bangkok, in 1958 [5, 6]. From late 2008 to the time of writing, the cumulative reported patient numbers reached a peak of over 20 000 cases. This infection is not uncommon, and sometimes must be included in the differential diagnosis of dengue. This paper shows a distinguishing manifestation of chikungunya, which may assist in the differential diagnosis from autoimmune inflammatory myopathies.

## 2. Case Report

A 21-year-old female from Yala Province, Southern Thailand, presented with high-grade fever during a vacation in Bangkok. She developed fever, polyarthralgia, and polymyalgia for 5 days; generalized erythematous rash was noted 1 day preadmission. She had no known underlying or familial disease, and no history of hair loss, oral ulcer, or photosensitivity. She denied traveling, or exposure to cattle, flooding, goat dairy products, unusual foods, or pets. Physical examination on admission showed temperature 38.6°C, BP 105/60 mmHg, PR 98/min, RR 24/min, mildly dehydrated lips, bilateral injected conjunctivae, bilateral palpable cervical lymph nodes ~0.5–1 cm in diameter, with predominant tenderness on the left postauricular nodes, and pain in the calves and joints of the extremities. Normal cardiovascular, pulmonary, and neurological findings were noted. Only a just-palpable, soft liver was noted on abdominal examination. CBC on admission (D0) is shown in Table 1. Her symptoms were alleviated by analgesics (for pain) and paracetamol (antipyretic). She was presumptively diagnosed with leptospirosis and began treatment with 2 gm/day of ceftriaxone.

However, more aggravated joint and muscle pain, with particular digital swellings at both hands and feet, were reported the following day. Physical examination supported this clinically, with puffy hands and feet with marked tenderness at the bilateral knuckles, including the metacarpophalangeal (MCP), distal and proximal interphalangeal (DIP and PIP) joints, and proximal muscle weakness in both upper and lower extremities. CBC and blood chemistry analysis the following day (D2) are shown in Table 1. Urine analysis found a yellow, clear fluid, specific gravity 1.020, pH 6.5, protein 0.0075 g/L, glucose negative, WBC 0-1/HPF, RBC 3–5/HPF, and epithelium 0-1/HPF. There was no evidence of hemolysis proven by direct microscopic blood film slide and Coomb's test negative. Serological tests were conducted for possible infections, including chikungunya disease. The IgM rapid chikungunya test and IgG using HAI both showed negative. IFA tests for scrub typhus and murine typhus showed negative for IgM and IgG. IFA tests for leptospiral antibody IgM and IgG were negative. However, serology for dengue infection and lyme disease were not tested because of not compatible clinical feature and endemicity of the diseases.

Three days later, the patient looked edematous and an erythematous rash had spread throughout her body. Physical examination revealed progressive painful joints and digits. She had to stay in bed because she was unable to get up from a prone position. CBC and blood chemistry results on day 5 are shown in Table 1. Elevated muscle enzyme was detected. ESR was 83 mm/hr. Urine analysis found a yellow clear fluid with specific gravity 1.015, pH 6.0, protein 0.0025 g/L, glucose negative, WBC 1-2/HPF, RBC 1-2/HPF, and epithelium 0-1/HPF. Hemocultures of 3 specimens showed negative. Secondary causes of polymyositis were assessed. Anti-HIV antibody was negative. Intravenous ceftriaxone was discontinued. High-dose steroid treatment was initiated to control inflammation, with 20 mg/day of dexamethasone, which was switched to 0.8 mg/kg/D oral prednisolone. After 3–5 days of steroid treatment, a significant clinical response was noted. She became afebrile, reported reduced pain in the joints, and resumed normal life after 1 week of treatment. CBC and blood chemistry results on D10 are shown in Table 1. ESR was 64 mm/hr. 

Serological testing confirmed acute chikungunya infection by EIA IgM 338 Units, IgG 15 Units. Serological tests were run each week, after the rapid tests. Other immunological laboratory investigations showed antinuclear antibody positive 1 : 320, speckled typed, anti-dsDNA negative, anti-SM negative, anti-JO-1 negative, VDRL nonreactive, and rheumatoid factor negative. Four to six weeks later, the patient improved markedly and resumed her normal activities. Steroid dosage was tapered off 10–15 mg/d every 2 weeks, and discontinued at 10 weeks. Antinuclear antibody levels were tested repeatedly and became negative 3 months after the onset of illness. The patient is now doing well.

## 3. Discussion

This case demonstrates an approach to the common problem of acute febrile illness in the Tropics. In differential diagnosis, it is necessary to explore the patient's medical history and assess exposure to risk, when no localized symptoms or signs are detected in a systemic infection. Laboratory screening investigations showed leukocytosis without thrombocytopenia coincidently with liver involvement in systemic illness. No specific liver or kidney injury was indicated. Leukocytosis can sometimes be found in early viral infections, and atypical bacterial infections in the tropics, such as leptospirosis, rickettsiosis, tuberculosis, including nontuberculous mycobacteria, and melioidosis, must be ruled out. Some animal-associated and toxin-producing bacterial infections should not be missed by extensive history review and clinical clues on the skin, eyes, mucosal, and musculoskeletal parts. The absence of thrombocytopenia, suggested that dengue or other hemorrhagic fever, and malaria, could probably be excluded. Followup complete-blood-count tests found atypical lymphocytes, supporting the suspicion of viral infection. Patient residence details can also point to a current outbreak of “Chikungunya infection” or simply, “CHIK.”

Chikungunya is screened by asking for possible symptoms in the list of diseases under investigation, including high fever, headache, myalgia, arthralgia, conjunctivitis, periorbital pain, joint swelling, erythematous rash, and petechial spots. Polyarthralgia and generalized myalgia were reported in nearly all cases (96 and 79%, resp.), while puffy hands and feet were found in only 19 and 16% of cases, respectively [7]. Serological tests can help establishing a diagnosis. Repeated laboratory tests are necessary in suspicious cases, even when the rapid screening test shows negative. Important clues are severe polymyositis and polyarthritis, especially of the small joints of the hands and the wrists, which are infrequently found in autoimmune rheumatism, apart from rheumatoid arthritis. A presentation of bilateral and symmetrical arthritis involving the hands and wrists, mimicking rheumatoid arthritis, may be resolved with seronegative rheumatoid factor and a medical history pointing to acute infection. Rheumatoid factor was reported positive in around 8–13% of chikungunya cases [7]. Followup investigations implied massive continuing inflammation, such as anemia with thrombocytosis, and elevated erythrocyte sedimentation rate (ESR). However, seropositivity for antinuclear antibodies was uncommonly detected in systemic infections, especially in a context of high titer dilution. Although several viral infections, such as Epstein-Barr virus (EBV), hepatitis virus, and cytomegalovirus (CMV), were reported to coexist with positive antinuclear antibodies [8] and the etiopathogenesis of autoimmune disease [9], chikungunya has not been found.

This is the first case report of autoimmune antibody detected during chikungunya infection and its disappearance postresolution. The existence of autoimmunity might explain the debilitating and prolonged aggravating musculoskeletal manifestation resulting from chikungunya infection in asymptomatic or early autoimmune disease. It remains to be elucidated why seropositivity lapsed with patient cure. In the current case, the patient's baseline immunostatus was unknown. It is probable that immune activation resulting from massive inflammation occurs simultaneously with viral cellular immune responses [10] and B-cell polyclonal activation against the host. Progression to clinical deterioration is triggered by viral replication, via proposed molecular mimicry [11]. There was only a temporal association between chikungunya infection and antinuclear antibodies in this interesting case report.

Good responses to high doses of steroids have been reported in many cases of arthropathy [12]. The actions of steroids in regulating gene function and inactivating the related proteins involving proinflammatory cytokines attenuate inflammatory myopathy. However, many deaths can be attributed to drugs (e.g., steroids) prescribed to treat symptoms [13]. The virus replicates in the endothelial cells, monocytes and macrophages in the lymph nodes, bone marrow, spleen, and liver. Viral mutation leads to new complications, such as hepatitis, cerebral meningitis, and coma, which have resulted in several deaths [14]. Proof of the benefits of corticosteroids and chloroquine would require a controlled study. Colchicine and chloroquine may be used as adjunct drugs.

## Figures and Tables

**Table 1:** Summary of laboratory investigations.

Investigations	D1	D2	D5	D10	M1	M3	Normal range
Hemoglobin (g/dL)	13.1	11.8	11.0	10.8	12.8	13.2	12–16
hematocrit (%)	39.2	34.8	31.5	32.3	37.1	39.8	37–47
MCV (fL)	90.7	91.2	86.2	89.2	91.3	92.3	82–96
WBC (×10^3^/*μ*L)	14.8	17.5	12.0	15.0	11.3	8.9	5.0–10.0
Neutrophils (%)	87	90	76	88, Band 3%	80	77	45–74, Band 0–4%
Lymphocytes (%)	4	4	6	5	14	21	16–45
Monocytes (%)	6	5	9	2	3	2	4–10
Eosinophils (%)	2	1	7	—	2	—	0–7
Basophils (%)	1	—	—	—	1	—	0–2
Atypical lymphocytes (%)	—	—	2	2	—	—	0–5
Platelets (×10^3^/*μ*L)	268	286	545	839	344	389	150–450
Urea nitrogen (mmol/L)	4.64	3.927	2.86	—	—	—	3.6–7.1
Creatinine (*μ*mol/L)	61.88	53.04	44.2	—	—	—	53–88
Glucose (mmol/L)	—	6.22	—	—	5.1	—	4.2–6.4
AST (*μ*kat/L)	1.75	2.15	2.02	0.92	0.52	0.55	0.1–0.66
ALT (*μ*kat/L)	2.33	2.28	1.95	1.42	0.46	0.56	0.1–0.66
Alkaline phosphatase (U/L)	119	101	92	—	—	—	50–136
Total bilirubin (*μ*mol/L)	13.9	10.94	8.55	—	—	—	5.1–17
Total protein (g/L)	70.8	68	66	—	—	—	64–82
Albumin (g/L)	35.1	36	30	—	—	—	34–50
Sodium (*μ*mol/L)	130	131	130	—	—	—	136–145
Potassium (*μ*mol/L)	3.65	3.8	3.5	—	—	—	3.5–5.0
Chloride (*μ*mol/L)	97	91	89	—	—	—	98–107
Bicarbonate (*μ*mol/L)	22	25	27	—	—	—	23–29
Creatinine kinase (*μ*kat/L)	—	—	16.35	10.22	1.22	1.04	0.17–1.17
Uric acid (*μ*mol/L)	—	—	208.18	—	—	—	90–360
